# Stakeholder Perceptions of Internet-Delivered Cognitive Behavior Therapy as a Treatment Option for Alcohol Misuse: Qualitative Analysis

**DOI:** 10.2196/14698

**Published:** 2020-03-03

**Authors:** Heather D Hadjistavropoulos, Kirsten M Gullickson, Shelley Adrian-Taylor, Andrew Wilhelms, Christopher Sundström, Marcie Nugent

**Affiliations:** 1 Online Therapy Unit Department of Psychology University of Regina Regina, SK Canada; 2 Department of Clinical Neuroscience Center for Psychiatric Research Karolinska Institutet Stockholm Sweden

**Keywords:** internet intervention, cognitive behavioral therapy, alcohol consumption, stakeholder participation, qualitative research, implementation science

## Abstract

**Background:**

Internet-delivered cognitive behavior therapy (ICBT) has been found to be effective for treating alcohol misuse in research trials, but it is not available as part of routine care in Canada. Recent recommendations in the literature highlight the importance of integrating perspectives from both patient and health care stakeholders when ICBT is being implemented in routine practice settings.

**Objective:**

This study aimed to gain an understanding of how ICBT is perceived as a treatment option for alcohol misuse by interviewing diverse stakeholders. Specifically, the objectives were to (1) learn about the perceived advantages and disadvantages of ICBT for alcohol misuse and (2) elicit recommendations to inform implementation efforts in routine practice.

**Methods:**

A total of 30 participants representing six stakeholder groups (ie, patients, family members, academic experts, frontline managers, service providers, and health care decision makers) participated in semistructured interviews. To be included in the study, stakeholders had to reside in Saskatchewan, Canada, and have personal or professional experience with alcohol misuse. Interviews were transcribed verbatim, anonymized, and analyzed using thematic analysis.

**Results:**

Stakeholders identified numerous advantages of ICBT for alcohol misuse (eg, accessibility, convenience, privacy, relevance to technology-based culture, and fit with stepped care) and several disadvantages (eg, lack of internet access and technological literacy, isolation, less accountability, and unfamiliarity with ICBT). Stakeholders also provided valuable insight into factors to consider when implementing ICBT for alcohol misuse in routine practice. In terms of intervention design, stakeholders recommended a 6- to 8-week guided program that uses Web-based advertising, point-of-sale marketing, and large-scale captive audiences to recruit participants. With regard to treatment content, stakeholders recommended that the program focus on harm reduction rather than abstinence; be evidence based; appeal to the diverse residents of Saskatchewan; and use language that is simple, encouraging, and nonjudgmental. Finally, in terms of population characteristics, stakeholders felt that several features of the alcohol misuse population, such as psychiatric comorbidity, readiness for change, and stigma, should be considered when developing an ICBT program for alcohol misuse.

**Conclusions:**

Stakeholders’ insights will help maximize the acceptability, appropriateness, and adoption of ICBT for alcohol misuse and in turn contribute to implementation success. The methodology and findings from this study could be of benefit to others who are seeking to implement ICBT in routine practice.

## Introduction

### Background

Alcohol misuse, defined as alcohol consumption that causes harm to the drinker, others, or society, is prevalent among Canadian adults, affecting 8% of individuals aged 15 years or older; this is higher than the world average of 5.1% [1]. Although there are numerous evidence-based treatments for alcohol misuse, it is known to be undertreated, with less than 15% of patients receiving services [2]. Many individuals refrain from seeking treatment because alcohol misuse is a particularly stigmatized mental disorder [3]. Compared with people with substance-unrelated mental disorders (eg, depression and schizophrenia), individuals with alcohol misuse are perceived as being more responsible for their condition, elicit more social rejection and negative emotions, are more likely to experience structural discrimination, and are less frequently regarded as mentally ill [3]. Other reasons for this treatment gap include client-related barriers (eg, embarrassment, desire to reduce drinking autonomously, and low motivation to change) and treatment-related barriers (eg, not believing that treatment will help, limited time available for treatment, and low proximity to treatment centers) [4].

Internet-delivered cognitive behavior therapy (ICBT) has emerged as an effective form of treatment that increases access to services by overcoming many barriers associated with face-to-face intervention [5]. ICBT is known to be more accessible, affordable, and convenient than traditional face-to-face care. Consequently, there is growing interest in offering ICBT in routine care [6]. ICBT typically involves users completing Web-based lessons on a weekly basis over the course of several months [7]. In recent years, several research groups have studied ICBT for alcohol misuse and promising treatment effects have been reported [8-11]. The overall goal of ICBT for alcohol misuse is typically behavioral change measured in terms of reduction of drinks consumed [11] as opposed to abstinence [9]. The design of ICBT treatments for alcohol misuse can differ in whether users work alone (ie, self-guided) or with a support person (ie, guided). Guided ICBT provides users with support in the form of email, Web-based chat, or brief telephone calls. Self-guided ICBT allows users to complete lessons independently with automated feedback or no contact at all. Previous research has provided preliminary evidence that guided ICBT results in greater reductions in alcohol consumption than self-guided ICBT [8,11].

Despite the mounting evidence supporting the efficacy of ICBT for alcohol misuse, it has yet to be implemented in routine care in Canada. Previous research has outlined numerous outcomes that should be addressed when implementing an intervention in a new setting [12,13]. Three outcomes that should be measured early in the implementation process are acceptability (ie, extent to which the intervention is agreeable, palatable, satisfactory, or useful), appropriateness (ie, perceived fit or compatibility of an intervention with its consumer’s needs), and adoption (ie, consumer’s intention to use the intervention) [12,13]. One of the recommended methods for assessing acceptability, appropriateness, and adoption is to gather the perspectives of a wide range of stakeholder groups (eg, health care providers and leaders, researchers, patients, and family members) by conducting semistructured interviews or focus groups before implementation [12,13]. Uptake of internet interventions, especially outside of research trials, is known to be far from satisfactory, and involving potential users in the development of such interventions is considered key in increasing use and impact [14]. Recent recommendations in the literature highlight the importance of integrating perspectives from both patient and health care stakeholders in implementation efforts of ICBT [15]; furthermore, several Canadian researchers have recently emphasized the importance of engaging stakeholders when developing and implementing electronic mental health interventions in Canada [16,17].

Considering acceptability, adoption, and appropriateness before implementation serves several purposes. First, it allows researchers to incorporate stakeholder feedback directly into the development of the ICBT program. Second, it ensures the intervention is patient oriented, ie, the intervention is “closely connected to the primary needs of the patients it is meant to serve” [17]. Third, it allows researchers to understand the local context within which the intervention will be delivered and enhance the appeal of the intervention [12,17]. Finally, and perhaps most importantly, it serves to promote implementation success and thus bolster service and treatment outcomes [13,18].

### Objectives

The purpose of this study was to interview diverse stakeholders who had personal or professional experience with alcohol misuse to gain a comprehensive understanding of how ICBT is perceived as a treatment option for alcohol misuse. Specifically, our objectives were to (1) learn about the perceived advantages and disadvantages of ICBT for alcohol misuse and (2) elicit recommendations to inform implementation efforts.

## Methods

### Geographical Context

This study was conducted in Saskatchewan, a landlocked province in Western Canada with a population of 1,098,352 [19]. Saskatchewan is geographically large (approximately 650,000 km^2^), with most residents living in the southern prairie half of the province rather than the sparsely populated northern forest region. Most of the population resides in two large cities with over 250,000 people each, but 35.6% of residents live in rural areas, which is substantially higher than the national average of 16.8% [19]. The average age of Saskatchewan residents is 39.1 years, with 64.8% of the population being aged between 15 and 64 years and 18.0% being 65 years or older [19]. Saskatchewan residents are primarily Euro-Canadian, but there is also a large portion of aboriginal peoples (16.3%; eg, First Nations, Metis, and Inuit) and a growing immigrant population (10.5%) [20].

### Health Care System and Clinical Setting

In Saskatchewan and across Canada, medical and mental health care are publicly funded and financed by the government through taxation [21]. Residents can access free mental health care via publicly funded hospitals and community mental health clinics. However, demand for public mental health services exceeds the supply of service providers available, so many residents turn to private mental health services, paid for out of pocket or by private health insurance [22]. The Online Therapy Unit at the University of Regina was created in part to improve Saskatchewan residents’ access to mental health care. Since its inception in 2010, the unit has been offering guided ICBT for depression and anxiety to residents of Saskatchewan, and educating mental health providers on the delivery of ICBT, and conducting research on ICBT in routine practice [23]. ICBT services are provided free of charge as a result of funding from the Saskatchewan Ministry of Health.

Of the more than 1000 clients who were screened for ICBT for depression and anxiety in the past year, approximately 20% self-reported alcohol misuse problems, suggesting that there is a need for ICBT for alcohol misuse in Saskatchewan. This anecdotal finding is supported by recent Census data, which suggest that 21.9% of people aged 12 years or older in Saskatchewan report engaging in heavy drinking at least once a month, compared with the Canadian average of 19.5% [19]. In addition, Saskatchewan has the third highest rate of impaired driving and the fifth highest rate of alcohol-related hospitalizations out of all provinces and territories in 2015, at 575 and 345 incidents per 100,000 people, respectively [24]. To meet the alcohol-related treatment needs of residents of Saskatchewan, the Online Therapy Unit is now planning on extending its services to include ICBT for alcohol misuse.

### Participants

Participants for this study were individuals from six stakeholder groups: patients (ie, individuals with direct lived experience), family members (ie, individuals with indirect lived experience), academic experts (ie, individuals who teach about or conduct research on alcohol misuse), frontline managers (ie, individuals who manage service providers), service providers (ie, individuals who work directly with patients), and health care decision makers (ie, individuals involved with government policy related to alcohol misuse). To be included in the study, stakeholders had to reside in Saskatchewan and have personal or professional knowledge of alcohol misuse. Our goal was to recruit numerous stakeholders from each group who could provide diverse perspectives on the topic of alcohol misuse. For example, we wanted stakeholders who could represent the perspectives of the urban and rural population and individuals with knowledge of different cultural perspectives. A priori, we hypothesized that a sample size of 20 to 30 stakeholders would be sufficient; however, we intended to continue conducting interviews until we were confident in the saturation of the data [25]. Stakeholders were primarily recruited via convenience sampling. Initial recruitment involved sending out an invitation to participate to alcohol misuse stakeholders known to the Online Therapy Unit (eg, previous ICBT participants and government officials involved in previous projects). Subsequently, snowball sampling was utilized, wherein stakeholders who were interviewed were asked to suggest other individuals who would have an interest in being involved in the study [26].

### Procedure

This study obtained approval from the institutional research ethics board. Interested participants were invited to take part in a semistructured interview with research staff members from the Online Therapy Unit in person or via telephone. Individual interviews were chosen over focus groups to maximize scheduling convenience for the stakeholders and because the stakeholders resided in geographically diverse locations across the province. One staff member (AW) was chosen to conduct interviews because of familiarity with alcohol misuse literature, whereas the other staff member (ST) was chosen because of experience in conducting qualitative interviews. The interview was comprised of 15 questions that provided insight into the acceptability, adoption, and appropriateness of ICBT for alcohol misuse in Saskatchewan (see Multimedia Appendix 1 for the interview guide). All interviews were audio-recorded with the permission of the participant. To ensure data quality, some interviews were attended by both staff members, with one member leading the interview and the other observing. Following such interviews, the researchers provided feedback to one another as an ongoing check to ensure that the interview process and interview questions allowed for the collection of open, honest, and impartial sharing of perspectives.

A total of 30 stakeholders were interviewed between October 2018 and January 2019. The participants who were interviewed represented a number of stakeholder groups (see Table 1). The interviews typically included one stakeholder at a time; however, there were three instances where two participants from the same organization preferred to be interviewed together. This resulted in 27 interviews in total. The interviews ranged in length from 18 to 67 min, with the average being 39 min. Five interviews were conducted in person and 22 by telephone. ST led 17 interviews and AW led 10. Eighteen interviews were attended by both staff members. Data collection was discontinued after 27 interviews (with 30 stakeholders) because all stakeholder groups had been adequately represented, and we were satisfied with our level of data saturation [25].

**Table 1:** Number of stakeholders by group (N=30).

Stakeholder type	Interviewed, n
Patients	7
Family members	3
Health care decision makers	7
Frontline managers	6
Service providers	4
Academic experts	3

### Data Analysis

Interview transcripts were transcribed verbatim, anonymized, and entered into QSR International’s NVivo 12 qualitative analysis software [27]. A descriptive, inductive approach to thematic analysis was utilized to identify themes within the interview data [28]. AW was responsible for reading each interview transcript closely to obtain an initial impression of the data and engage in open coding, wherein basic codes that represent each unit of meaning were derived. Subsequently, identified codes were discussed with ST to ensure content coverage. Finally, after AW comprehensively coded all the data, several members of the research team (HH, KG, MN, and ST) came together to sort the individual codes into meaningful themes.

## Results

### Stakeholder Opinions

Stakeholders from all groups (eg, patients, family members, academic experts, frontline managers, service providers, and health care decision makers) resoundingly endorsed the implementation of ICBT for alcohol misuse in Saskatchewan. Moreover, they provided a wealth of information to be considered when developing an ICBT program for alcohol misuse. The results are discussed below, and illustrative stakeholder quotes are provided to enhance reader understanding.

### Perceived Advantages and Disadvantages of Internet-Delivered Cognitive Behavior Therapy for Alcohol Misuse

When discussing the immense need for ICBT for alcohol misuse in Saskatchewan, stakeholders highlighted some perceived advantages and disadvantages of an ICBT approach to the treatment of alcohol misuse. The advantages and disadvantages are described in Tables 2 and 3, respectively, along with example stakeholder quotes.

**Table 2:** Perceived advantages of internet-delivered cognitive behavior therapy for alcohol misuse. ICBT: internet-delivered cognitive behavior therapy.

Advantages	Description	Example quote
Accessibility	ICBT allows clients with alcohol use who live in rural and remote locations to access care that is otherwise limited.	“For people who are in the rural areas I think it [ICBT for alcohol misuse] is hugely advantageous, because getting an addictions counselor in rural areas can be really challenging.” [Stakeholder #12, Patient]
Convenience	ICBT allows clients who are limited by cost, transportation, or time constraints to access care.	“There are individuals that have obligations with work or parenting where they are not able to necessarily attend programming during some of the traditional hours that they’re offered or even outside of traditional hours, right? So being able to access a program in their home is definitely a need.” [Stakeholder #6, Frontline Manager]
Privacy	ICBT allows clients to participate in treatment from a private location, which reduces the chances of experiencing stigma related to alcohol misuse.	“[ICBT for alcohol misuse] is more private, it's not like they have to walk into a public meeting and say “Hi, I'm an alcoholic.” That can be very intimidating.” [Stakeholder #24, Family Member]
Relevance to technology-based culture	ICBT is congruent with today’s technology-based culture.	“Our world is living in that direction [toward technology], so it would make sense to have a service [like ICBT] that's delivered in a way that lots of people can access. Technology is just part of life.” [Stakeholder #3, Service Provider]
Fit with stepped care	ICBT fits within a stepped-care model, wherein clients with less severe alcohol misuse can use ICBT, leaving more intensive in-person services for those with more severe alcohol misuse.	“[ICBT] is really the starting part of a step-care approach so that not everybody needs to go to addiction services. Sometimes it’s just a matter of gathering information for themselves or for others and I think it could potentially have a role in prevention. It could potentially have an impact on wait times too*.”* [Stakeholder #22, Health Care Decision Maker]

**Table 3:** Perceived disadvantages of internet-delivered cognitive behavior therapy for alcohol misuse. ICBT: internet-delivered cognitive behavior therapy.

Disadvantages	Description	Example quote
Requires internet access and technological literacy. ICBT: internet-delivered cognitive behavior therapy.	ICBT requires clients to have access to the internet and the knowledge and ability to use a computer, tablet, or mobile phone, which may prohibit some individuals from participating.	“A lot of individuals don’t have access to Internet. Oftentimes we see a population who are disadvantaged so they may not have a stable house where they can access a computer to access online ICBT. They may not have access to a phone where they can get it on their mobile.” [Stakeholder #2, Health Care Decision Maker]
May foster isolation	ICBT may reinforce isolation that can be associated with alcohol misuse by allowing clients to engage in treatment without leaving home.	“It [addiction] is about disconnection; disconnection with ourselves, disconnection with others. How do you make it [an ICBT program for alcohol misuse] in a way that is not allowing clients to further isolate?” [Stakeholder #25, Academic Expert]
Less accountability	The Web-based nature of ICBT could allow clients to be less accountable for their actions than they would be if they had face-to-face meetings with their service provider or peers.	“It’s easier not to be accountable to a computer than it is to an addiction counsellor or an AA group.” [Stakeholder #28, Health Care Decision Maker]
Unfamiliarity with ICBT	Clients might not be aware that ICBT is an available treatment option for alcohol misuse.	"Unfamiliarity with this whole resource [ICBT] could be one [disadvantage].” [Stakeholder #15, Frontline Manager]

### Factors to Be Considered When Implementing Internet-Delivered Cognitive Behavior Therapy for Alcohol Misuse

**Figure 1:**
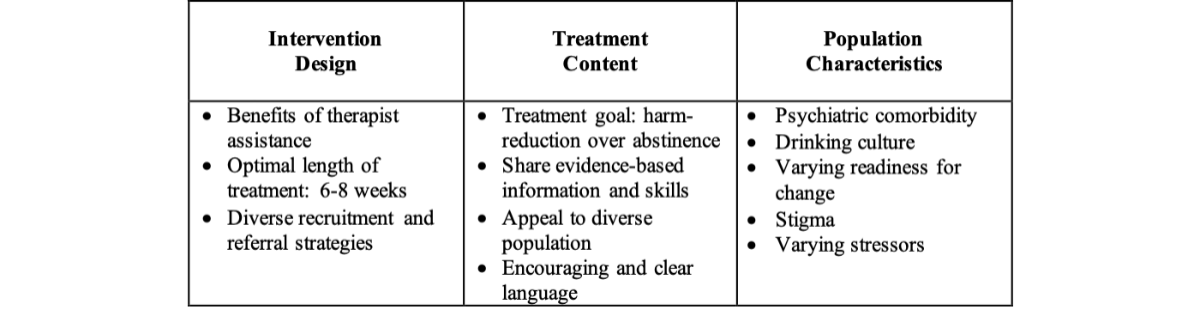
Factors to consider when implementing internet-delivered cognitive behavior therapy for alcohol misuse.

Stakeholders provided valuable insight into factors to consider when implementing ICBT for alcohol misuse in Saskatchewan. Three themes emerged from the analysis: (1) intervention design, (2) treatment content, and (3) population considerations (Figure 1).

#### Intervention Design

##### Benefits of Guided Internet-Delivered Cognitive Behavior Therapy

The majority of stakeholders suggested that a guided ICBT program would be beneficial because it would allow for greater support, increase accountability, and provide clients with someone to connect with. In terms of the best method of guidance, stakeholders felt that offering email support in combination with telephone follow-ups would be important. However, several stakeholders indicated that it might be helpful for clients to be able to choose the level and type of support that fits their needs (ie, guided vs self-guided, email vs telephone contact). When discussing the value of guidance, one stakeholder explained:

I think it [guided ICBT] is a happy medium between having to go see a therapist and having to do it all on your own.

Stakeholder #26, Patient

##### Optimal Length of Treatment: 6 to 8 Weeks

When stakeholders were asked about the ideal length of treatment, responses ranged from 6 to 12 weeks. The majority of stakeholders suggested a 6- to 8-week time frame. Some recommended a longer treatment length of 10 to 12 weeks, but other stakeholders expressed concern that clients would be less likely to commit to a longer treatment period. A number of stakeholders suggested that the length of treatment may need to be altered depending on a number of factors (eg, interest and alcohol misuse severity).

##### Diverse Recruitment and Referral Strategies

Stakeholders felt that advertising to potential clients on the Web using social media and Web advertisements would be most effective. The value of recruiting via social media was described by one stakeholder who commented:

[Facebook] is where everyone is going these days; that’s where you're guaranteed to get the best reach. Facebook is really targeted these days in that you can select people who live in Regina who are between the ages of 14 and 35 who enjoy drinking [...] you can target ads to every single group [related to alcohol], so you can pick out exactly who you need and who's going to be successful in your program just with one click of a button.

Stakeholder #26, Patient

Several additional strategies were also recommended, including point-of-sale advertising (ie, advertisements in places where alcohol is sold or consumed such as liquor stores, restaurants, bars, and sporting events) and media advertising (eg, newspaper and radio). With regard to referrals, stakeholders recommended creating a professional referral network to increase coordination of care among alcohol misuse service providers. Specifically, they suggested the exchange of information and referrals with counselors, physicians, community health clinics, social workers, and public education or advocacy groups. 

#### Treatment Content

##### Treatment Goal: Harm Reduction Over Abstinence

A few stakeholders discussed abstinence and harm reduction approaches to the treatment of alcohol misuse. Stakeholders generally held the perspective that harm reduction was a more appropriate approach, particularly for people who are drinking at mild-to-moderate levels. However, there was also recognition that clients can choose abstinence within a harm reduction model if their goal was to eliminate alcohol consumption altogether. One stakeholder explained:

I think that [the idea of abstinence] turns people off, the idea that you can’t have something anymore, then that... You know, you almost want it even more because you can’t have it.

Stakeholder #20, Patient

##### Sharing Evidence-Based Information and Skills

A number of stakeholders emphasized the importance of including evidence-based information and skills in the ICBT program. They underscored the value of teaching clients what constitutes alcohol misuse and about the low-risk drinking guidelines. They also recommended providing education about the effects of alcohol, especially the physical effects and how to deal with withdrawal. They suggested using language such as *experts say* and *other participants have said* when introducing new skills to underscore that the treatment content is based on existing evidence. One stakeholder highlighted this subtheme when they said:

People often don’t know how to moderate their drinking. They don’t understand what a standard drink is. There is a need for [education] about that.

Stakeholder #21, Frontline Manager

##### Appeal to Diverse Populations

Stakeholders spoke of the importance of gaining the trust of potential clients by delivering content that demonstrates understanding and sensitivity to the diversity that exists among individuals with alcohol misuse in terms of culture, age, and socioeconomic status. Stakeholders indicated that content such as photos, wording, and case stories must be relatable and relevant to individuals from various cultures. Stakeholders also spoke of alcohol misuse as a cross-generational issue and recommended that the content appeal to adults across the life span. Stakeholders finally recommended that the program content be sensitive to socioeconomic diversity, as people struggling with alcohol misuse are likely to range from those who live in poverty to those who are wealthy. This point was illustrated by one stakeholder who commented:

We [individuals with alcohol misuse] come from all walks of life. I mean, you could be a doctor, you could be a janitor, you could be a 16-year old kid, you could be a 90-year old grandmother. It could be anybody... like I said, it’s all walks of life so it [alcohol misuse] crosses all boundaries.

Stakeholder #11, Patient

##### Encouraging and Clear Language

All stakeholders spoke to the importance language and messaging would play, not only in drawing potential clients to the Online Therapy Unit website but also in keeping them engaged once participating in the program. Stakeholders advised against language or information that is confrontational, difficult to understand, and stigmatizing or has the potential to trigger feelings of hopelessness. Instead, stakeholders spoke of the need to use encouraging, nonjudgmental, and simple language. One stakeholder explained the importance of removing stigmatizing language:

I would say if you’re looking to hook in people who are moderate drinkers, I would avoid the term “alcoholic,” because I think some people would look at that and think, “Well, I’m not an alcoholic, I’m not a drunk, that’s not me.”

Stakeholder #28, Frontline Manager

#### Population Considerations

##### Psychiatric Comorbidity

The majority of stakeholders highlighted the challenge of providing treatment for alcohol misuse in the context of comorbid conditions. Many spoke of the strong connection between alcohol misuse and other mental health conditions, such as anxiety, depression, and trauma. It was suggested that an ICBT program for alcohol misuse take into account and possibly even address comorbid psychiatric conditions. One stakeholder described the link between alcohol misuse and mental illness:

The drinking is just a symptom of deeper emotional issues and so a lot of people aren’t aware of that in the beginning part of their journey, they think alcohol is the problem, not the fact that they have years of trauma.

Stakeholder #12, Patient

##### Drinking Culture

The vast majority of stakeholders described the impact of drinking culture on alcohol consumption. They explained that alcohol consumption is typically considered an acceptable, normal activity that goes hand in hand with the day-to-day social activities. Thus, they warned that reducing or eliminating alcohol consumption might contribute to feelings of social isolation. They stressed the importance of the ICBT program acknowledging societal drinking culture and preparing clients for reducing their alcohol consumption within this context.

##### Varying Readiness for Change

Several professional stakeholders described the importance of the ICBT program considering potential clients’ readiness to change their drinking behavior. Relatedly, a number of patients and family members discussed denial or lack of recognition of a drinking problem as a reality for some individuals with alcohol misuse. Stakeholders indicated that it may be valuable to conduct an assessment and provide feedback to allow potential clients to understand their level of alcohol misuse and its negative effects; they suggested this may reduce ambivalence about following through with an ICBT program. The importance of addressing readiness for change was described by one stakeholder who stated:

That level of motivation to want to change, sometimes that’s just not there, so I think getting an accurate assessment of where they are in that stages of change model would be helpful, but even within that, we know that it’s cyclical and people can revert back to contemplation or pre-contemplation where that motivation to follow through on a program like this just isn’t there.

Stakeholder #17, Frontline Manager

##### Stigma

Several stakeholders talked about the shame and stigma associated with alcohol misuse being a significant barrier to seeking help. In particular, a number of patients and family members spoke of the feeling of not wanting anyone to know they had a problem because if that had become known it may have had negative consequences. Stakeholders suggested the ICBT program be aware of the enormous stigma faced by individuals with alcohol misuse.

##### Varying Stressors

Stakeholders spoke of the importance of recognizing the variety of stressors that can be experienced by individuals who misuse alcohol. They noted that alcohol consumption can be not only a source of stress but also a way of coping with stress. One stakeholder discussed stress as a social determinant of health:

It’s important to acknowledge the social determinants of health that have led to or support that abuse. [...] You'd have to talk about what's your housing situation, where are living, do you have a job, etc. [...] Because if you're an alcoholic who lives at home with a family and you have a job, you're in a much, much different situation than someone who's living in a shelter.

Stakeholder #26, Patient

## Discussion

### Principal Findings

This study aimed to interview stakeholders who had personal or professional experience with alcohol misuse to understand their perceptions of ICBT as a treatment option for alcohol misuse in routine practice. Along with a resounding endorsement for implementation, stakeholders shared their perceptions about the advantages and disadvantages of using ICBT to treat alcohol misuse and made numerous recommendations related to intervention design, intervention content, and the population being treated.

The perceived advantages and disadvantages identified by stakeholders were consistent with previous ICBT research [5]. Understanding stakeholders’ perceptions of these advantages and disadvantages is helpful as we proceed with implementation efforts. The perceived advantages of ICBT for alcohol misuse—accessibility, convenience, privacy, relevance to technology-based culture, and fit with stepped-care—can be emphasized to potential clients and referral sources during recruitment. One advantage that seems especially relevant to the alcohol misuse population relates to privacy, given the strong stigma experienced by individuals with alcohol misuse [3]. Accordingly, the ability to participate in ICBT from a private location might be of particular value to potential clients with alcohol misuse and thus should be strongly emphasized.

In contrast, the perceived disadvantages of ICBT for alcohol misuse—requires internet access and technological literacy, may foster isolation, less accountability, and unfamiliarity with ICBT—represent areas to be acknowledged and mitigated as implementation proceeds. Perhaps the most addressable disadvantage is unfamiliarity with ICBT. When implementing ICBT for alcohol misuse in routine practice, significant effort can be made to inform potential clients and referral sources about the nature and availability of the treatment. Other disadvantages might be mitigated by incorporating certain features into the ICBT program for alcohol misuse; eg, guided ICBT likely has greater potential to lessen clients’ isolation and improve accountability compared with a self-guided approach. In addition, designing a program that is downloadable and can be accessed through a variety of technological devices (eg, computer, tablet, or mobile phone) may benefit those without a home computer or a consistent internet connection.

In terms of intervention design, stakeholders indicated that a guided program with both email and telephone correspondence would be preferable as it would allow for greater support, increased accountability, and human connection. This suggestion is supported by two previous studies that found guided ICBT programs for alcohol misuse to be both more engaging and more effective than self-guided ICBT [8,11]. With regard to treatment content, stakeholders recommended that the treatment goal of the program should be reduction of, rather than abstinence from, alcohol consumption. This treatment goal is also supported by previous research showing the benefits of harm reduction approaches relative to abstinence [29]. Finally, the population characteristics noted by the stakeholders are supported by existing literature. In particular, stakeholders felt that several common features of the alcohol misuse population, such as psychiatric comorbidity and readiness for change, should be taken into account when developing an ICBT program for alcohol misuse. It is well known that alcohol misuse is often comorbid with other mental health disorders [30] and that readiness for change is an important predictor of successful behavior change [31].

### Strengths and Limitations

This study has several important strengths. First, to our knowledge, this is the first study related to implementation of ICBT for alcohol misuse in routine care. It should be stated that the advantages and disadvantages identified in the stakeholder interviews were in line with what has been previously identified in the literature on internet interventions [5]. However, there were several important recommendations provided by the stakeholders that are unique for the alcohol misuse population. These include the promotion of harm reduction rather than abstinence and the importance of taking into account factors such as psychiatric comorbidity and readiness to change when developing the ICBT program. Second, this study is patient oriented, ensuring that a new ICBT program for alcohol misuse will closely reflect the needs and expectations of the alcohol misuse population, thereby maximizing the probability of implementation success. The fact that we included stakeholders from various groups and regions of Saskatchewan ensures that our results represent a wide range of perspectives. Moreover, our confidence in our level of data saturation makes us certain that interviewing additional stakeholders would have resulted in diminishing returns.

Despite its strengths, this study also has several limitations. First, although we expect the findings of this study to be largely applicable to other geographic locations, the inclusion of stakeholders exclusively from Saskatchewan may hamper generalizations to other geographical contexts. Second, with regard to the sample, there may be a selection bias as some stakeholders may have had initial favorable attitudes toward ICBT. For example, 8 out of 30 stakeholders (27%) had professional or personal experience with other ICBT courses being offered by the Online Therapy Unit. Relatedly, the use of snowball sampling increases the risk that stakeholders referred like-minded individuals to participate. In terms of the interview guide, it is conceivable that the semistructured nature of the interview influenced the stakeholders’ responses. Specific interview questions may have implicitly encouraged stakeholders to speak to certain topics and not to others. Moreover, the choice to conduct individual interviews rather than focus groups could be regarded as a limitation, as a focus group format could have resulted in broader discussion, enhancing the richness of the results. Finally, as with all qualitative research, it is possible that the researchers’ knowledge and previous experience with ICBT and the treatment of alcohol misuse implicitly influenced how the interview content was interpreted. As described by Braun and Clarke [28], “data are not coded in an epistemological vacuum.”

### Future Directions

The recognizable next step is to move forward with the development and implementation of ICBT for alcohol misuse in routine practice. This will include continuing to assess acceptability, appropriateness, and adoption once the intervention is implemented. Moreover, other implementation outcomes such as feasibility, fidelity, and implementation cost can be measured in addition to service outcomes (eg, effectiveness, efficiency, and timeliness) and client outcomes (eg, satisfaction, functioning, and symptomatology). Attempts should be made to replicate the findings from this study among stakeholders in other geographical locations and practice settings. We encourage researchers interested in implementing ICBT for alcohol misuse (or any other condition) to use our interview guide and analytic procedure as a model. Finally, as ICBT becomes more common in routine care, there is likely to be ongoing value in taking a patient-oriented approach, whereby patient partners are engaged in all aspects of program development and evaluation. Past research suggests that adopting a patient-oriented approach ensures treatment is tailored to the needs of the clients it is meant to serve, which can improve satisfaction and patient outcomes [32].

### Conclusions

Conducting semistructured interviews with 30 stakeholders who had personal or professional experience with alcohol misuse provided valuable insights that will inform the development of an ICBT program for alcohol misuse in routine practice. In terms of intervention design, stakeholders recommended a 6- to 8-week guided program. With regard to treatment goals, stakeholders recommended that the program focus on harm reduction rather than abstinence. In terms of population characteristics, stakeholders believed that several features of the alcohol misuse population, such as psychiatric comorbidity and readiness for change, should be taken into account when developing an ICBT program for alcohol misuse. The feedback provided by stakeholders will be used to bolster acceptability, appropriateness, and adoption and in turn contribute to implementation success. The methodology and findings from this study can serve as an example to others who are seeking to deliver ICBT in routine practice.

## Appendix

Multimedia Appendix 1 Interview guide.

## Abbreviations

ICBT: internet-delivered cognitive behavior therapy

## References

[1] Poznyak V, Rekve D, World Health Organization (2018). Global status report on alcohol and health 2018.

[2] Cohen E, Feinn R, Arias A, Kranzler HR (2007). Alcohol treatment utilization: findings from the National Epidemiologic Survey on Alcohol and Related Conditions. Drug Alcohol Depend.

[3] Schomerus G, Lucht M, Holzinger A, Matschinger H, Carta MG, Angermeyer MC (2011). The stigma of alcohol dependence compared with other mental disorders: a review of population studies. Alcohol Alcohol.

[4] Saunders SM, Zygowicz KM, D'Angelo BR (2006). Person-related and treatment-related barriers to alcohol treatment. J Subst Abuse Treat.

[5] Andersson G, Titov N (2014). Advantages and limitations of internet-based interventions for common mental disorders. World Psychiatry.

[6] Titov N, Dear B, Nielssen O, Staples L, Hadjistavropoulos H, Nugent M, Adlam K, Nordgreen T, Bruvik KH, Hovland A, Repål A, Mathiasen K, Kraepelien M, Blom K, Svanborg C, Lindefors N, Kaldo V (2018). ICBT in routine care: a descriptive analysis of successful clinics in five countries. Internet Interv.

[7] Andersson G (2016). Internet-delivered psychological treatments. Annu Rev Clin Psychol.

[8] Blankers M, Koeter MW, Schippers GM (2011). Internet therapy versus internet self-help versus no treatment for problematic alcohol use: a randomized controlled trial. J Consult Clin Psychol.

[9] Kiluk BD, Devore KA, Buck MB, Nich C, Frankforter TL, LaPaglia DM, Yates BT, Gordon MA, Carroll KM (2016). Randomized trial of computerized cognitive behavioral therapy for alcohol use disorders: efficacy as a virtual stand-alone and treatment add-on compared with standard outpatient treatment. Alcohol Clin Exp Res.

[10] Riper H, Kramer J, Smit F, Conijn B, Schippers G, Cuijpers P (2008). Web-based self-help for problem drinkers: a pragmatic randomized trial. Addiction.

[11] Sundström C, Gajecki M, Johansson M, Blankers M, Sinadinovic K, Stenlund-Gens E, Berman AH (2016). Guided and unguided internet-based treatment for problematic alcohol use - a randomized controlled pilot trial. PLoS One.

[12] Hermes ED, Lyon AR, Schueller SM, Glass JE (2019). Measuring the implementation of behavioral intervention technologies: recharacterization of established outcomes. J Med Internet Res.

[13] Proctor E, Silmere H, Raghavan R, Hovmand P, Aarons G, Bunger A, Griffey R, Hensley M (2011). Outcomes for implementation research: conceptual distinctions, measurement challenges, and research agenda. Adm Policy Ment Health.

[14] Fleming TM, de Beurs D, Khazaal Y, Gaggioli A, Riva G, Botella C, Baños RM, Aschieri F, Bavin LM, Kleiboer A, Merry S, Lau HM, Riper H (2016). Maximizing the impact of e-therapy and serious gaming: time for a paradigm shift. Front Psychiatry.

[15] Wilhelm S, Weingarden H, Ladis I, Braddick V, Shin J, Jacobson N (2020). Cognitive-behavioral therapy in the digital age: presidential address. Behav Ther.

[16] Lal S (2019). E-mental health: promising advancements in policy, research, and practice. Healthc Manage Forum.

[17] McGrath P, Wozney L, Rathore SS, Notarianni M, Schellenberg M (2018). Toolkit for e-Mental Health Implementation.

[18] (2013). Canada Health Infoway.

[19] (2017). Statistics Canada.

[20] (2014). Saskatchewan Ministry of Health.

[21] Marchildon GP, Hadjistavropoulos HD, Koocher GP, Hadjistavropoulos T, Hadjistavropoulos H (2014). Health psychology within the health-care system. Fundamentals Of Health Psychology.

[22] Sunderland A, Findlay LC (2013). Statistics Canada.

[23] Hadjistavropoulos HD, Nugent MM, Alberts NM, Staples L, Dear BF, Titov N (2016). Transdiagnostic internet-delivered cognitive behaviour therapy in Canada: an open trial comparing results of a specialized online clinic and nonspecialized community clinics. J Anxiety Disord.

[24] Perreault S Statistics Canada.

[25] Saunders B, Sim J, Kingstone T, Baker S, Waterfield J, Bartlam B, Burroughs H, Jinks C (2018). Saturation in qualitative research: exploring its conceptualization and operationalization. Qual Quant.

[26] Johnson TP (2014). Wiley Online Library.

[27] NVivo Qualitative Data Analysis Software.

[28] Braun V, Clarke V (2006). Using thematic analysis in psychology. Qual Res Psychol.

[29] Marlatt G, Witkiewitz K (2002). Harm reduction approaches to alcohol use: health promotion, prevention, and treatment. Addict Behav.

[30] Grant B, Goldstein R, Saha T, Chou P, Jung J, Zhang H, Pickering R, Ruan J, Smith S, Huang B, Hasin D (2015). Epidemiology of DSM-5 alcohol use disorder: results from the National Epidemiologic Survey on Alcohol and Related Conditions III. JAMA Psychiatry.

[31] Williams EC, Kivlahan DR, Saitz R, Merrill JO, Achtmeyer CE, McCormick KA, Bradley KA (2006). Readiness to change in primary care patients who screened positive for alcohol misuse. Ann Fam Med.

[32] (2011). Canadian Institutes of Health Research.

