# Supplementary material for: Stakeholder Perceptions of Internet-Delivered Cognitive Behavior Therapy as a Treatment Option for Alcohol Misuse: Qualitative Analysis
Source: JMIR Ment Health. 2020 Mar 3;7(3):e14698. doi: 10.2196/14698 (PMC7078623; doi:10.2196/14698)
Supplement: Multimedia Appendix 1 [file mental_v7i3e14698_app1.docx]

**Multimedia Appendix 1: Interview guide**.

**General**

1. In your opinion, is there a need for an internet-delivered cognitive behaviour therapy (ICBT) program targeting alcohol misuse in Saskatchewan? Yes/No *(Probes: Why? Tell me more about that.)*
2. What do you feel are the major services/information that already exist for this population (people experiencing mild to moderate problems with alcohol)? *(Probes: In-person services? Print material? Where can it be accessed from? How useful is this service/information? Any examples of what specifically makes it useful/not useful? What are some limitations to this service/information?*)
3. What should we know about the alcohol misuse population when developing an ICBT program? (*Probes: What is unique about this group?*)
4. How likely would you/your family/friends/clients be to use an ICBT program for alcohol misuse?
5. What are some barriers for use of an ICBT program among this population? *(Probes: Think about people you know. What would prevent them from using it? What would make them more likely to use it?)*

**Coordinated model**

1. What services should be coordinated with an ICBT program for alcohol misuse? *(Probes: Who should know about us so that they can refer to us? Who should we consider referring to? Who should we partner with?)*

**Recruitment strategies**

1. What types of recruitment strategies do you think would be most effective for promoting ICBT for alcohol misuse? *(Probes: What communication methods? Who can help spread the word? What has worked in the past?)*
2. What language or messages are appealing to people experiencing problems with alcohol? *(Probes: What language is effective? What language should be avoided?)*

**Design and format**

1. What are your thoughts on length of ICBT treatment for alcohol misuse? (*Probes: How many weeks is ideal?*)
2. What are your thoughts on the level of support needed among this population? (*Probes: therapist support, self-help only, peer support, frequency of support*)
3. If therapist-support is offered, what method of therapist-client communication would you suggest? *(Probes: telephone, email, other? Why that method?)*

**Other**

1. Who else do you think we should be interviewing? *(Probes: Who specifically? Can you provide us with their contact information?)*
2. Is there anything else you would like to share that we haven’t already talked about?
